# Prevalence of COVID-19 in health professionals and occupational psychosocial risks

**DOI:** 10.47626/1679-4435-2021-625

**Published:** 2021-04-30

**Authors:** Mafalda Sousa-Uva, Antonio Sousa-Uva, Florentino Serranheira

**Affiliations:** 1 Departamento de Epidemiologia, Instituto Nacional de Saúde Doutor Ricardo Jorge, Lisboa, Portugal; 2 Centro de Investigação em Saúde Pública, Lisboa, Portugal; 3 Departamento de Saúde Ocupacional e Ambiental, Escola Nacional de Saúde Pública, Universidade NOVA de Lisboa, Lisboa, Portugal

**Keywords:** worker health, public health, COVID-19, health professionals

## Abstract

**Introduction:**

Health professionals who provide clinical care are exposed to patients potentially infected by the severe acute respiratory syndrome coronavirus 2 SARS-CoV-2), namely physicians and nurses; consequently, these professionals face higher risks of infection.

**Objectives:**

This study aimed to describe the prevalence of coronavirus disease 19 (COVID-19) cases among health professionals and the frequencies of risk factors and psychosocial risk.

**Methods:**

This is a cross-sectional study targeted at health professionals working in Portugal during the current COVID-19 pandemic. Data were obtained through a self-administered questionnaire available online at the websites of medical and nursing boards, among other sources. We performed a univariate analysis, calculating absolute and relative frequencies, and a bivariate analysis with a Pearson’s chi-squared test.

**Results:**

We studied 4,212 health professionals, of which 36.7% (n = 1,514) worked in areas dedicated to the treatment of sick or suspected COVID-19 patients. Of these, 2.11% tested positive for SARS-CoV-2. Among all participants, 76.7% and 79.1% presented moderate to severe levels of fatigue and anxiety, respectively. Fatigue levels were significantly higher in professionals working in areas dedicated to the treatment of patients with COVID-19 (80.5% p = 0.01), but this difference was not observed regarding anxiety (79.5% p = 0.681).

**Conclusions:**

The percentage of health professionals who tested positive for SARS-CoV-2 was 2.11%. The reported high levels of fatigue and anxiety should determine a better protection of the health and safety of those who provide health care in the current pandemic.

## INTRODUCTION

Coronavirus disease 2019 (COVID-19) is a severe acute respiratory syndrome caused by coronavirus 2 (SARS-CoV-2) that was described for the first time in the city of Wuhan, China, at the end of 2019.^1-4^ The disease quickly spread worldwide and was declared a public health emergency of international interest by the World Health Organization (WHO) on January 30, 2020. By the end of February 2020, most cases were already being reported outside China, more significantly in Europe. In fact, on June 7, 2020, Europe had 2,052,235 cases of the disease, and Russia was the most affected country in absolute numbers (458,689) while Portugal reported 34,351 cases.^5^

Portugal reported its first 2 officially confirmed cases of COVID-19 on March 2, 2020. Many countries, such as the United Kingdom,^6^ were confident in their preparation for fighting this pandemic, which in many cases was revealed not to be true; one example was the availability of mechanical ventilation equipment, which was insufficient in various occasions.^7^ Moreover, difficulties inherent to the discrepancy between the offer and demand of personal protective equipment (PPE) have been highlighted. Soon, person-to-person transmission and direct or indirect (contact with surfaces) contagion through small droplets released by speaking, coughing, or sneezing were recognized.^1,8,9^

Health professionals providing clinical care, such as physicians and nurses, are thus exposed to potentially infected patients, consequently facing a higher risk of infection.^10^ In Portugal, as in other countries, the risks of contagion drove organizational measures in hospitals and Health Center Groups (Agrupamentos de Centros de Saúde [ACES]) that differentiated the health care provided to suspected COVID-19 patients from that provided to patients with other pathologies.

Cases of infection among health professionals, in Portugal, have varied from 10% to 15% of all COVID-19 cases, even with access to PPE; this illustrates the high contagiousness of SARS-CoV-2. On May 21, 2020, the Secretary of State for Health reported that out of 29,912 cases of the disease, 3,317 were health professionals: 480 of these were physicians and 1,088 were nurses.^11^ This proportion of infected health professionals revealed that the microbiological agent was an occupational risk factor and COVID-19 was an occupational disease among health care providers.^12^ This proportion of infected professionals occurred despite rigorous access to PPE and the immediate creation of areas dedicated to confirmed or suspected cases (named areas dedicated to COVID-19) both in hospital emergency services and in primary health care, aiming to protect users and accompanying persons, as well as health professionals.

The risk factors related to the current pandemic are not limited to the specific risk of contracting COVID-19, since many other aspects related to providing care in a pandemic context increase risk situations. Among other aspects, psychosocial risk factors^13^ are worth mentioning due to the increase in professional demands and workload; the need to perform while wearing uncomfortable PPE; the increase in working hours or, in many cases, the separation from family members.

## METHODS

The present study aimed to describe the prevalence of COVID-19 (positive cases for SARS-CoV-2) among health professionals during the first pandemic wave. We also intended to describe the frequency of some risk factors and occupational risks, such as symptoms and other psychosocial aspects. We designed an observational, analytic, cross-sectional study aimed at all health professionals working in Portugal between April 2 and April 10, 2020.

Data were collected through a self-administered online questionnaire built using the Google Forms platform, which was made available at the websites of Escola Nacional de Saúde Pública and medical and nursing boards, among other sources. At the end of 2018, Portugal had 53,657 active physicians and 73,650 active nurses registered in the respective boards.^14^ Subsequently, the questionnaire was promoted through e-mail lists and social media (mostly professional). Our sample is thus a convenience sample that reflects the voluntary adherence to filling the questionnaire within the referred timeframe.

The questionnaire considered sociodemographic data (sex, age group, region); data on COVID-19 infection (self-monitoring of body temperature twice a day and of the presence/absence of symptoms; whether the participant was a suspected COVID-19 case, was under active or passive surveillance, was tested for COVID-19, and which was the result; and time between suspicion and testing); and data on occupational risk factors (professional group, workplace, and PPE availability). This last topic was especially focused on risk factors and occupational psychosocial risks, namely the self-perception of fatigue and anxiety measured by the Portuguese version of the Hospital Anxiety and Depression Scale^15^. This scale classifies scores of 0 to 7 as “no anxiety symptoms,” 8 to 10 as “mild symptoms,” 11 to 14 as “moderate anxiety symptoms,” and 8 to 10 as “severe anxiety symptoms.”^16^

Statistical analyses included a descriptive univariate analysis performed by calculating absolute and relative frequencies stratified by the variables of interest. A bivariate statistical analysis was also performed, verifying the independence between variables (work in an area dedicated to COVID-19 suspected or confirmed cases and other variables) through a Pearson’s chi-squared (χ2) test. The statistical significance level was 5%, and the analysis was performed using SPSS.

## RESULTS

This study included 4,212 health professionals, which in 2018^17^ corresponded to 4.1% of all personnel working at hospitals and health care centers of the National Health Service of mainland Portugal. Most respondents (75.8%) were female and aged between 30 and 49 years (61.6%). Regarding professional groups, 31.9% were physicians, 29.4% were nurses, 17.9% were diagnostic and therapeutics technicians, 2.6% were medical auxiliaries, and 18.2% belonged to other health professions such as pharmacists, dietitians, psychologists, and laboratory professionals (Table 1)

**Table 1:** Characteristics of health professionals in this study

Characteristic	n	%
Sex		
Female	3,112	75.8
Male	993	24.2
Age group (years)		
20-29	461	11.2
30-39	1,330	32.2
40-49	1,211	29.4
50-59	771	18.7
60 and older	353	8.6
Region		
Alentejo and Algarve	191	4.6
Centro	538	13.0
Lisboa and Vale do Tejo	1,678	40.7
Norte	1,664	40.3
Autonomous Regions	55	1.3
Professional group		
Medical auxiliary	107	2.6
Nurse	1,215	29.4
Physician	1,315	31.9
Diagnostic and therapeutics technician	737	17.9
Other	752	18.2
Workplace		
ACES	1,403	34.0
Continued care	40	1.0
Hospital	1,853	44.9
More than one option	185	4.5
Other	645	15.6
Works with confirmed or suspected COVID-19 cases		
Yes	1,514	36.7
No	2,612	63.3
Performs daily self-monitoring		
Yes	2,720	65.9
No	1,406	34.1
Is a suspected COVID-19 case		
Yes	562	13.6
No	3,564	86.4
Is a suspected case under surveillance		
Active	217	38.6
Passive	345	61.4
Is a suspected case tested for COVID-19		
Yes	412	73.3
No	150	16.7
Time between COVID-19 suspicion and testing (hours)		
Less than 24	118	28.6
24-48	103	25.0
48-72	70	17.0
More than 72	121	29.4
Test result (confirmed COVID-19 case)		
Positive	64	16.2
Negative	332	83.8
PPE availability (0-6 scale)		
Unavailable (0)	102	2.5
Insufficient (1 and 2)	1,161	28.2
Moderate (3 and 4)	1,965	47.7
Sufficient (5 and 6)	898	21.1
		
Fatigue (0-6 scale)		
Absent (0)	222	5.4
Low (1 and 2)	742	18.0
Moderate (3 and 4)	2,231	54.1
Severe (5 and 6)	931	22.6
Anxiety (Hospital Anxiety and Depression Scale)		
Normal	90	2.2
Possible	772	18.7
Moderate	2,033	49.3
Severe	1,231	29.8

ACES = Health Care Center Groupings; COVID-19 = coronavirus disease 2019; PPE = personal protective equipment.

Most health professionals work at hospitals (44.9%) and ACES (34.0%) in the Lisboa and Vale do Tejo region (40.7%) and in the Norte region (40.3%). Notably, 36.7% (n = 1,514) of the respondents work in areas dedicated to treating sick or suspected COVID-19 patients, namely at primary care (35.9%), hospitals (53.6%), and 5% work in both (Table 2).

**Table 2:** Characteristics of health professionals who worked or not with suspected or confirmed COVID-19 cases and results of the chi-squared test for the relationship between working with patients with COVID-19 and other variables considered in the analysis

Characteristic	Health professionals who did not work with suspected or confirmed COVID-19 cases (n = 2,612)		Health professionals who worked with suspected or confirmed COVID-19 cases (n = 1,514)		χ^2^ test
	n	%	n	%	χ^2^ (p-value)
Sex					
Female	1,989	76.6	1,123	74.5	2.2 (0.141)
Male	609	23.4	384	25.5	
Age group (years)					
20-29	292	11.2	169	11.2	48.3 (< 0.001)
30-39	795	30.4	535	35.3	
40-49	739	28.3	472	31.2	
50-59	508	19.4	263	17.4	
60 and older	278	10.6	75	5.0	
Region					
Alentejo and Algarve	127	4.9	64	4.2	61.3 (< 0.001)
Centro	374	14.3	164	10.8	
Lisboa and Vale do Tejo	1,128	43.2	550	36.3	
Norte	939	35.9	725	47.9	
Autonomous Regions	44	1.7	11	0.7	
Professional group					
Medical auxiliary	57	2.2	50	3.3	86.1 (< 0.001)
Nurse	744	28.5	471	31.1	
Physician	783	30.0	532	35.1	
Diagnostic and therapeutics technician	443	17.0	294	19.4	
Other	585	22.4	167	11.0	
Workplace					
ACES	859	32.9	544	35.9	225.0 (< 0.001)
Continued care	36	1.4	4	0.3	
Hospital	1,041	39.9	812	53.6	
More than one option	109	4.2	76	5.0	
Other	567	21.7	78	5.2	
Performs daily self-monitoring					
Yes	1,625	62.2	1,095	72.3	43.6 (< 0.001)
No	987	37.8	419	27.7	
Is a suspected COVID-19 case					
Yes	323	12.4	239	15.8	9.53 (< 0.001)
No	2,289	87.6	1,275	84.2	
Is a suspected case under surveillance					
Active	120	37.2	97	40.6	0.68 (0.411)
Passive	203	62.8	142	59.4	
Is a suspected case tested for COVID-19					
Yes	235	72.8	177	74.1	0.120 (0.73)
No	88	27.2	62	25.9	
Time between suspicion and testing (hours)					
Less than 24	52	22.1	66	37.3	11.64 (0.009)
24-48	62	26.4	41	23.2	
48-72	44	18.7	26	14.7	
More than 72	77	32.8	44	24.9	
Test result (confirmed COVID-19 case)					
Positive	32	14.3	32	18.6	1.34 (0.247)
Negative	192	85.7	140	81.4	
PPE availability (0-6 scale)					
Unavailable (0)	71	2.7	31	2.0	70.6 (< 0.001)
Insufficient (1 and 2)	817	31.3	334	22.7	
Moderate (3 and 4)	1,244	47.6	721	47.6	
Sufficient (5 and 6)	480	18.3	409	27.6	
Fatigue (0-6 scale)					
Absent (0)	161	6.2	61	4.0	23.12 (0.010)
Low (1 e 2)	508	19.4	234	15.4	
Moderate (3 e 4)	1,382	52.9	849	56.1	
Severe (5 e 6)	561	21.5	55	24.4	
Anxiety (Hospital Anxiety and Depression Scale)					
Normal	58	2.2	32	2.1	1.51 (0.681)
Possible	494	18.9	278	18.4	
Moderate	1,298	49.7	735	48.5	
Severe	762	29.2	469	31.0	

ACES = Health Center Groups; PPE = personal protective equipment

Among all health professionals, 13.6% (n = 562) were suspected COVID-19 cases, of which 15.8% (n = 239) work in areas dedicated to patients with COVID-19. Out of all respondents, 34.1% (n = 1,406) did not perform daily self-monitoring of body temperature and symptoms. This is especially important for 27.7% (n = 419) of health professionals, who worked in areas dedicated to patients with COVID-19 (p < 0.001) (Table 2). Considering suspected cases among health professionals, only 38.6% were under active surveillance by the responsible bodies (occupational health services and/or health authorities). Regarding those who worked in areas dedicated to patients with COVID-19, only 40.5% of the respondents were actively surveilled (by occupational health services and/or health authorities) and no significant differences were observed in comparison with those who worked in other areas (p = 0.411).

Around three-quarters of the professionals were tested for SARS-CoV-2, and of these, 28.6% was tested within the first 24 hours and 29.4% was only tested more than 72 hours after first suspicion. Among professionals who work in areas dedicated to patients with COVID-19, the number of respondents tested within the first 24 hours was significantly higher (37.3%, p = 0.009) (Table 2). In this study, 1.55% (64/4,126) of the participants tested positive for SARS-CoV-2 (16.2% of the suspected cases and 2.11% of the professionals working in COVID-19-dedicated areas). We did not observe a significant difference between the numbers of positive cases among workers of areas dedicated to patients with COVID-19 or not (p = 0.247).

PPE availability was classified as sufficient for 21.1% of the professionals and as insufficient for 28.2% of them. Professionals from COVID-19-dedicated areas provided slightly better answers regarding PPE availability and considered it sufficient in 27.6% of the cases (p < 0.001).

Fatigue was referred to as moderate to severe by more than three-quarters of the professionals (76.7%), and it was significantly higher in professionals working in COVID-19-dedicated areas (80.5%, p = 0.01). Anxiety levels reported by health professionals stood out negatively. More than three-quarters presented moderate to severe anxiety levels (79.1%). Nevertheless, the levels of anxiety reported by professionals of areas dedicated to the treatment of sick or suspected COVID-19 patients were similar (79.5%, p = 0.681) to those observed in other health care sectors.

## DISCUSSION

The strength of the SARS-CoV-2 pandemic wave and its high contagiousness characteristics gave rise, within the global scientific community, to an unprecedented response, and many research teams have dedicated much of their activities to better understanding the natural history of COVID-19. Soon it became clear that health professionals were particularly exposed^7^ and this exposure was not limited to the contact with the infectious agent, but also included other risk factors such as those of psychosocial nature. In fact, the increased workload, uncertainty, and initial difficulties with PPE availability in many countries, as well as distancing from family members as a protective measure, are good examples of the need to manage occupational risks also within the scope of the mental health of health care professionals.

In this study, 1.55% (n = 64) of the respondent health professionals tested positive for SARS-CoV-2 (2.11% of those working in areas dedicated to treating sick or suspected COVID-19 cases). In Portugal, by June 15, 2020, 0.35% of the population had tested positive for SARS-CoV-2.^11^ The frequency of COVID-19 cases among health professionals thus seems to be 4 times higher than that in the overall Portuguese population. It is important to note that this comparison should not be performed due to methodological issues, since this study did not consider a representative sample of the health professional population. However, this result seems to be revealing of COVID-19 as a specific occupational disease risk. We also observed a lack of studies on the frequency of confirmed COVID-19 cases among health professionals,^18^ which hampers the discussion of these results. Nevertheless, a cross-sectional study in the Netherlands described a prevalence of 6.4%,^19^ whereas a longitudinal study in China (Wuhan) verified an incidence of 38.9%^20^; both results were greater than those observed in this study.

Among the results obtained in this study, we highlight that more than one-third of the health professionals did not perform the required daily self-monitoring (namely the daily recording of body temperature and presence of symptoms)^( 21)^; this should be critical to the decision of whether or not going to work in order to interrupt the chain of infection of this disease. This behavior may be related to high professional demands, which do not leave time for such an apparently simple measure that could avoid new contagions.

PPE availability was also not considered satisfactory by more than 1 in 4 respondents. This reveals that, under these work circumstances, some health professionals may eventually feel unsafe and have the perception of risking their health and consequently that of their family members, which may contribute to an increase in anxiety levels.

Fatigue and anxiety affected more than three-quarters of the respondent professionals (76.7% and 79.1%, respectively), indicating that greater effort should be applied to protecting the health of care providers. Studies performed in China indicated a lower occurrence of moderate to severe levels of anxiety: 12.3%^22^ and 24%^23^. However, these studies used the Generalized Anxiety Disorder 7-item (GAD-7) scale, which is not applicable specifically to measuring anxiety in health professionals. Moreover, cultural differences may also justify these result discrepancies. It is important to note that a study performed in Portugal with health professionals in a non-pandemic context observed anxiety levels of 61.5%.^24^ This study, despite not being comparable due to methodological issues among other reasons, revealed an increase of almost 20%.

Anxiety levels were not significantly different between health professionals working with patients confirmed or suspected for COVID-19 and those who provided care in areas not dedicated to these patients. The same was not verified in studies performed in China.^18^ This discrepancy may be explained by various factors, such as the greater professional demands that those who were not on the front lines also had to face in Portugal. These were eventually associated to a greater workload considering the care of patients not related to COVID-19 due to a lack of professionals who were recruited to the front lines for fighting this pandemic. Similarly, the uncertainty regarding the progression of this pandemic and the enormous attention in the media received by this subject may have influenced all citizens living in Portugal.

Finally, it is important to mention some of the limitations of the present study, namely the selection bias and the impossibility of generalizing results to the whole health professional population in Portugal since this is a convenience sample. In addition, we mention the information bias brought by the self-administered questionnaire and the courtesy bias associated to the fact that respondents were health professionals participating in a study that was also conducted by health professionals.

In conclusion, the fatigue and anxiety levels of health professionals in Portugal in the context of the first COVID-19 pandemic wave were high (76.7% and 79.1%, respectively). In this regard, it is imperative to remember that the current response to the pandemic aims to protect the life and health of citizens, and this is especially true when considering the life and health of care providers, who constitute a critical group and are essential for fighting this pandemic. We highlight that the circumstances of epidemiological approaches are not the main objective of our intervention but represent a decisive means for better understanding the characteristics that are essential for protecting the life and health of citizens.

The protection of the health and safety of health professionals thus represents a crucial (if not decisive) measure for managing the current pandemic in the perspective of health care; it should be object of wider and better attention by everyone, namely by those responsible for defining and implementing public policies that fight occupational risks.
